# Genetic characterization of Tibetan pigs adapted to high altitude under natural selection based on a large whole-genome dataset

**DOI:** 10.1038/s41598-024-65559-3

**Published:** 2024-07-24

**Authors:** Lingyun Zhang, Yanbin Zhu, Yue Ren, Linna Xu, Xuanbo Liu, Xuebin Qi, Ting Jiao, Guangming Sun, Haiyu Han, Jian Zhang, Fengbo Sun, Yanan Yang, Shengguo Zhao

**Affiliations:** 1 College of Animal Science and Technology, Gansu Agricultural University, Lanzhou, China; 2 Academy of Agriculture and Animal Husbandry Sciences, Institute of Animal Husbandry and Veterinary Medicine, Lhasa, China; 3 State Key Laboratory of Genetic Resources and Evolution, Chinese Academy of Sciences, Kunming Institute of Zoology, Kunming, China; 4 College of Grassland Science, Gansu Agricultural University, Lanzhou, China; 5 The Animal Husbandry Station in Changdu, Changdu, China; 6 The Beast Prevention Station in Gongbujiangda County, Linzhi, China; 7 The Animal Husbandry Station in Tibet Autonomous Region, Lhasa, China

**Keywords:** Tibetan pigs, High-altitude adaptation, WGS, SNP, Natural selection, Population genetics, Animal breeding, Genetic markers, Bioinformatics, Genomic analysis

## Abstract

The Qinghai-Tibet Plateau is a valuable genetic resource pool, and the high-altitude adaptation of Tibetan pigs is a classic example of the adaptive evolution of domestic animals. Here, we report the presence of Darwinian positive selection signatures in Tibetan pigs (TBPs) using 348 genome-wide datasets (127 whole-genome sequence datasets (WGSs) and 221 whole-genome single-nucleotide polymorphism (SNP) chip datasets). We characterized a high-confidence list of genetic signatures related response to high-altitude adaptation in Tibetan pigs, including 4,598 candidate SNPs and 131 candidate genes. Functional annotation and enrichment analysis revealed that 131 candidate genes are related to multiple systems and organs in Tibetan pigs. Notably, eight of the top ten novel genes, *RALB*, *NBEA*, *LIFR*, *CLEC17A*, *PRIM2*, *CDH7*, *GK5* and *FAM83B,* were highlighted and associated with improved adaptive heart functions in Tibetan pigs high-altitude adaptation. Moreover, genome-wide association analysis revealed that 29 SNPs were involved in 13 candidate genes associated with at least one adaptive trait. In particular, among the top ten candidate genes, *CLEC17A* is related to a reduction in hemoglobin (HGB) in Tibetan pigs. Overall, our study provides a robust SNP/gene list involving genetic adaptation for Tibetan pig high-altitude adaptation, and it will be a valuable resource for future Tibetan pig studies.

## Introduction

Tibetan pigs (TBPs) are indigenous domestic animals that have lived on the Qinghai-Tibet Plateau for more than 2,500 generations^1^, allowing natural selection to accumulate adaptive SNPs to help them cope with high-altitude environments^2^. The genetic adaptation of TBPs to high altitudes is viewed as a typical example of the adaptive evolution of domestic animals^3–6^, which exhibit phenotypes, including developed capillaries, thinner alveolar septa, thicker alveolar septa, and larger and stronger hearts^7,8^. Hypoxia poses a major barrier to life and has been shown to regulate redox homeostasis in large yellow croaker^9^ and induce brain injury in cynomolgus monkeys^10^; however, a number of indigenous animals, including TBPs, yaks, and Tibetan sheep, are well adapted to hypoxic enviroments^5,11,12^. Studies have indicated that high-altitude adaptation (HAA) in domestic animals is mediated by the mutation of *EPAS1*, which acts on the hypoxia pathway (HIF)^13^. Two studies have suggested that *EPAS1* contributes to HAA in TBPs by reducing the hemoglobin (HGB) concentration^3,5^. In human studies, it has also been found that Tibetans at high altitudes have lower hemoglobin^14^, this is related to reproductive rate and athletic ability^15^. However, other studies have hypothesized that lower hemoglobin concentrations are the result of larger hemoglobin mass and larger plasma volume^16^. It is possible that different species have different adaptive regulation of the plateau^17^, these phenotypes are regulated by host genes. A total of 226 signaling genes likely under natural selection in TBPs have been reported in previous studies^3,4,6,18–22^ (Supplementary Tables 1, 2). Although numerous studies have been performed over the past several decades, the genetic basis of high-altitude adaptation to TBPs has still not been elucidated due to severely poor phenotypic and whole genome data.

Among the reasons for this situation are that current studies lack comprehensiveness in two ways: (1) The adaptive phenotypes are unknown, and few studies have investigated the differences in phenotypes between TBPs and lowlander pigs (LDPs) at high altitudes. (2) There was no large-scale whole genome sequencing (WGS) data available to reveal the relationships between positively selected genes and phenotypes. In this study, we used the WGS data system to screen for positive selection of TBPs and analyzed the differential phenotypes of TBPs living at different altitudes on the Tibetan Plateau. This study provides a high-confidence SNP/gene set and a set of potential adaptive phenotypes, revealing the genetic characteristics of Tibetan pigs adapting to low oxygen environments during natural selection.

## Method

### Sample collection and DNA extraction

We collected 221 blood samples (146 from TBPs, 35 from YKXs (Yorkshire pigs), and 40 from Duroc pigs) in the Tibet Autonomous Region at different altitudes (Supplementary Table 1). All animal samples were collected with the consent of the farmers and approved by the Ministry of Science and Technology of the People's Republic of China (Approval number: 2006–398). All animal experiments involved in this study were approved by the Animal Ethics Committee of Gansu Agricultural University (gau-eth-ast-2021-023). We have complied with Animal Research: Reporting In Vivo Experiments (ARRIVE) protocol at submission. We used an automatic hemocyte analyzer (GRT-6008, Jinan Glitter Technology Co., Ltd.) to perform blood physiological measurements. Genomic DNA from 221 TBP individuals was extracted from blood samples using a QIAGEN DNeasy Blood & Tissue Kit. DNA concentrations were measured with a NanoDrop 2000 (Thermo Fisher Scientific), and the DNA was sent to a company (KANGPUSENG) for sequencing (compass No. 1).

### Phenotypic data analysis

First, we calculated the mean and the sample distribution for each phenotype. We considered the phenotypic score to be an outlier and removed points for which the score was greater than or less than the mean ± 3 SD (standard deviation). Next, we conducted a difference analysis for each phenotype. We used a *t*-test to compare the differences between TBPs and LDPs (YKX, Duroc) and used the R function of false discovery rate (FDR) for multiple correction. FDR < 0.05 was considered to indicate statistical significance. Moreover, we analyzed the correlation between phenotype and altitude and found that HGB in YKXs increased with altitude, while TBPs showed a significant blunt effect on TBPs. Finally, we conducted correlation analysis for 9 phenotypes by R with multiple adjustments by FDR, and HGB, hematocrit (HCT) and red blood cell counts (RBCs) showed significant correlations (Fig. 2D).

### SNP calling

FastaQC was used to perform the quality control of the raw data. Per-individual sequence reads were aligned using the ‘mem’ algorithm “bwa mem –M -R @RG\tID:name\tSM:name” in the Burrows–Wheeler Algorithm (BWA) (http://biobwa.sourceforge.net/bwa.shtml) to the reference *Sus_scrofa* (Sscrofa 11.1) and then converted to Binary Alignment/Map (BAM) format, sorted by genomic position and indexed using samtools (https://www.htslib.org/doc). The GATK4 Mark Duplicates module was used to mark the potential duplicate reads inherited from the library construction step for chromosome-wise duplicate marking per individual, in which amplified PCR errors could introduce incorrect variants in variant calling. The haplotype Caller module in GATK was used for SNP and INDEL calling chromosome-wise simultaneously for each sample, as it is more accurate to call variants in some special regions with the de novo local assembly method, especially when calling INDELs. For population-based analyses, the GATK Genotype GVCF module was applied to the GVCFs generated from the previous step to call the variants for each chromosome of the combined TBP, YKX, and Duroc datasets. The chromosome-wis raw variants were combined with genome-wide raw variants for population-based variant VCFs and individual-based GVCFs. We then conducted hard filtering by GATK (filter expression “QD < 2.0 || MQ < 40 || FS > 60.0 || SOR > 3.0 || MQRankSum < −12.5 || ReadPosRankSum < −8.0”). Ultimately, 17,486,881 SNPs were identified for downstream analysis.

### Imputation

The two levels at which individuals and loci were detected were used for quality control of the chip data. At the individual level, we removed individuals with missing data greater than 5% and heterozygosity greater than or less than 3 times the standard deviation (SD), and individuals with significant differences in population structure were also removed by principal component analysis (PCA). Two samples were removed due to failed missing- heterozygosity, and 17 (10 YKX and 7 TBPs) samples were excluded because of admixture. At the locus level, we removed the following variants: (1) variants showing a significant deviation from Hardy–Weinberg equilibrium (*p* < 0.000001); (2) variants with an excessive missing genotype rate (MGR > 0.05); and (3) variants with minor allele frequency below the provided threshold of 0.03 (maf < 0.05). A total of 44,295 SNPs passed quality control (QC) and were used or imputation.

Compared with other pig breeds, TBPs lack a good haplotype reference; therefore, we constructed a TBP haplotype reference from the WGS data we collected. Immut2 (https://mathgen.stats.ox.ac.uk/impute) was used to carry out the imputation of chip data, and the SNPs with impute-info < 0.3 were removed. Ultimately, 306,497 SNPs passed QC as a clean dataset set for downstream Genome-Wide Association Studies (GWAS) analysis.

### Principal component analysis (PCA)

**Figure 1:**
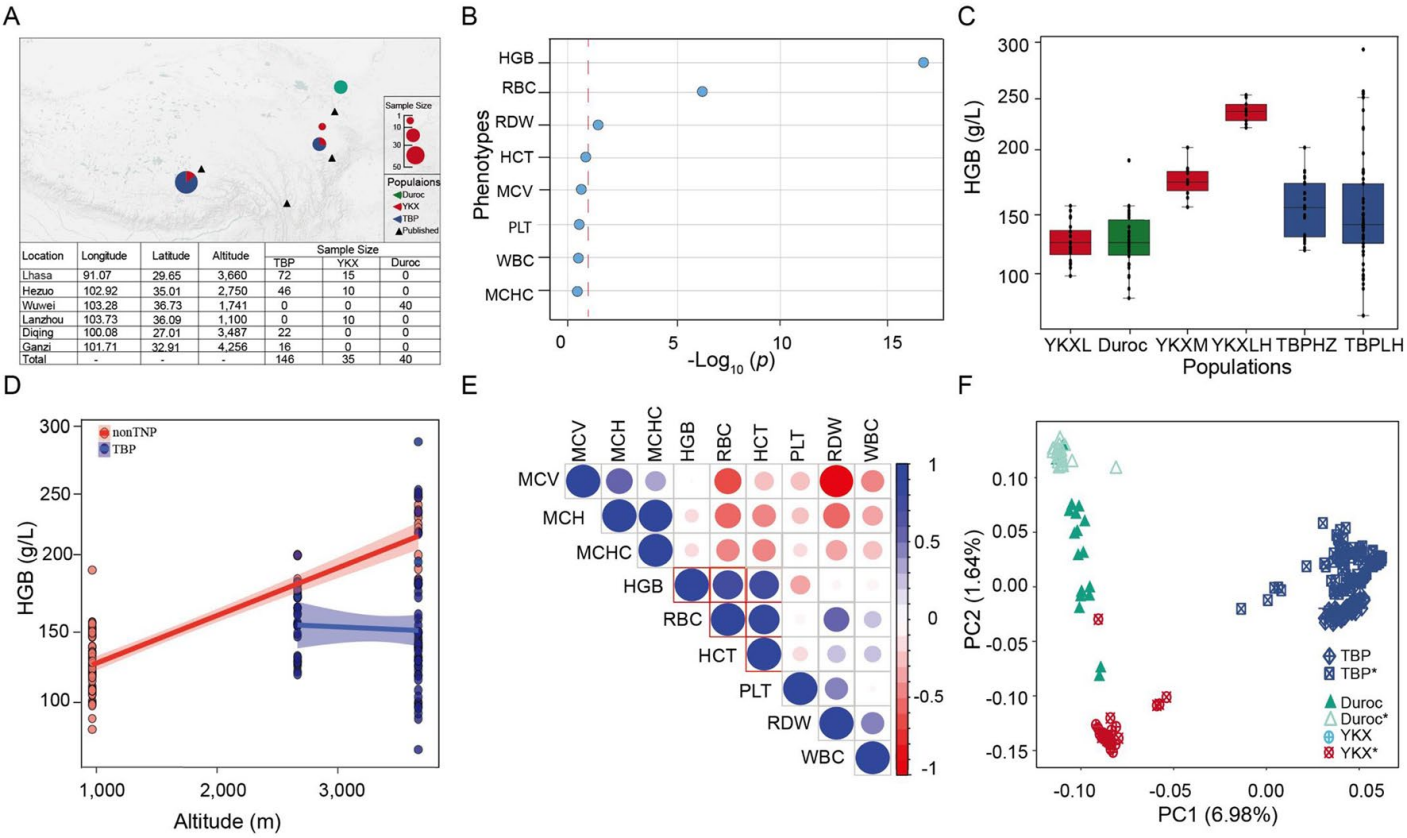
The data used in this study. (**A**) Geographical distribution of samples in our study. The circles in the figure show the samples collected in this study, the triangles were downloaded from published TBPs, the red sector represents the YKX, the blue sector represents TBPs, and the green sector represents Duroc pigs. The circle sizes represent the sample sizes, and the map was generated Google Maps. (**B**) Differential analysis of TBPs vs YKX blood phenotypes. We calculated the phenotypic differences between TBPs and YKX at the same altitude. FDR for multiple corrections was performed, *p* < 0.05 was considered to indicate statistical significance, and the x-axis is −log_10_ (p value). (**C**) Comparison of differences in hemoglobin among TBPs and other populations. (**D**) Correlations between the TBP and YKX altitudes and hemoglobin levels. (**E**) Correlations among different blood indices in TBPs. (**F**) Principal component analysis (PCA). There were no differences in population structure between TBPs and published TBPs (blue), TBPs*: the published TBPs; YKX*: the published YKX; Duroc*, the published Duroc pigs.

Principal component analysis (PCA) was performed at the individual level using EIGENSOFT (version 3.0) to investigate fine-scale population structure and individual genetic affinities. We performed a series of PCAs by gradually removing ‘outliers’ based on a plot of the first and second principal components (PCs) and reanalyzing the remaining samples based on the same set of SNP markers (Fig. 1F).

### Identification of genomic positive selection regions

The QC of the samples and SNPs was performed on 127 (69 TBPs, 25 YKXs and 33 Duroc pigs) WGS datasets, and 26 samples (9 TBPs, 9 YKXs, 8 Duroc pigs) were excluded due to population admixture. After SNP level QC, 9,197,506 SNPs remained and were included in the positive selection statistics. To identify the positively selected regions in the TBP genome, we calculated the frequency-based (*F*_ST_), haplotype-based (iHS, XPEHH, DiHH, nSL) and maximum likelihood-based XPCLR. After QC, for samples with YKX < 20, to obtain accurate allele frequencies, we used Duroc pigs as a reference population (*n* = 25). We used five of the calculated values (*F*_ST_, iHS, XPEHH, DiHH, nSL) as the CMS input files to perform the CMS analysis, and the calculated formulas were obtained from a previous study^23^.

We conducted LD-based clumping with an index variant *p* value threshold greater than significant Content Management System (CMS) scores (top 1‰ (8.82) across the whole genome), an SP2 column *p* value threshold greater than significant CMS scores (in the top 1% across the whole genome), an r^2 threshold of 0.2, and a clump kb radius of 500 kb. Candidate regions to be selected had greater than 5 SNPs according to the SP2 threshold, and the genes with the peak SNPs were identified as TBP candidate positive selection genes (TCSGs) (Supplementary Table 3). Finally, we obtained 131 TCSGs (Supplementary Table 4).

### Annotation of SNPs

We annotated SNPs with SNPEFF (http://snpeff.sourceforge.net/), VEP (Variant Effect Predictor, http://www.ensembl.org/info/docs/tools/vep), and ANNOVAR (http://www.openbioinformatics.org/annovar). For those SNPs (i.e., the high-impact SNVs), we took their intersection as the final trusted dataset set and obtained 69 TBP-enriched missenses (Supplementary Table 5).

### Gene-based KEGG and GO enrichment analyses

To understand TCSG functions at the genetic level, we performed Kyoto Encyclopedia of Genes and Genomes (KEGG)^24,25^and Gene Ontology (GO) enrichment analyses using gProfiler (https://biit.cs.ut.ee/gprofiler/gost) (Supplementary Table 6).

### Association analysis

Plink 2.0 (https://s3.amazonaws.com/plink2-assets/alpha2/) was used for genome-wide association analysis of the TBPs adaptive phenotypes of the TBPs. The linear additive model revealed that age, sex, and altitude were concomitant factors. Permulation tests were performed 100,000 times for p adjustment (Supplementary Table 7).

### Linking the TCSGS to organs/systems

To establish possible relationships between TCSGs and organs, the pig eQTL database was downloaded from pigQTLdb (https://www.animalgenome.org/QTLdb/app), and we mapped the eQTL positions to our TCSGs using bedtools (https://bedtools.readthedocs.io/en/latest). Fisher’s precision test was used to detect significant enrichment in TBP organs/systems.

## Results

### Phenotypic analysis

To understand the adaptive phenotypes of TBP to HAA, we measured the physiological indices of 221 blood samples collected from pigs (146 TBPs, 35 YKXs and 40 Duroc pigs) living at different altitudes (1,000–4,000 m) on the Qinghai-Tibet Plateau in China (Fig. 1A). After strict QC was performed, we analyzed the phenotypes. Among the nine phenotypes, hemoglobin (HGB), red blood cell counts (RBCs), and red blood cell distribution width (RDW) were significantly different between TBPs and YKXs at high altitudes (*p* < 0.05, FDR < 0.001) (Fig. 1B; Supplementary Fig. 1A–H). HGB was a drastically fluctuated in TBPs and showed a blunted response to hypobaric hypoxia (Fig. 1C, D). Phenotypic analysis also revealed strong correlations HGB, RBCs and hematocrit (HCT) (Fig. 1E).

We performed genome-wide genotyping (compass No. 1) on 221 individuals and acquired 48,000 clean SNPs. We conducted principal component analysis (PCA) on all SNPs in 3 published populations (69 TBPs, 16 YKXs and 25 Duroc pigs)^3–5,18–20^. Our TBPs significantly overlapped with those of reported TBPs and were clearly stratified by PC1. The genetic relatedness of the TBPs was consistent with results from the WGS data of previous studies^3^^,^^5^. Overall, these results suggested that our TBPs were representative of indigenous TBPs (Fig. 1F).

### Detecting the genome-wide adaptation signals of TBPs

**Figure 2:**
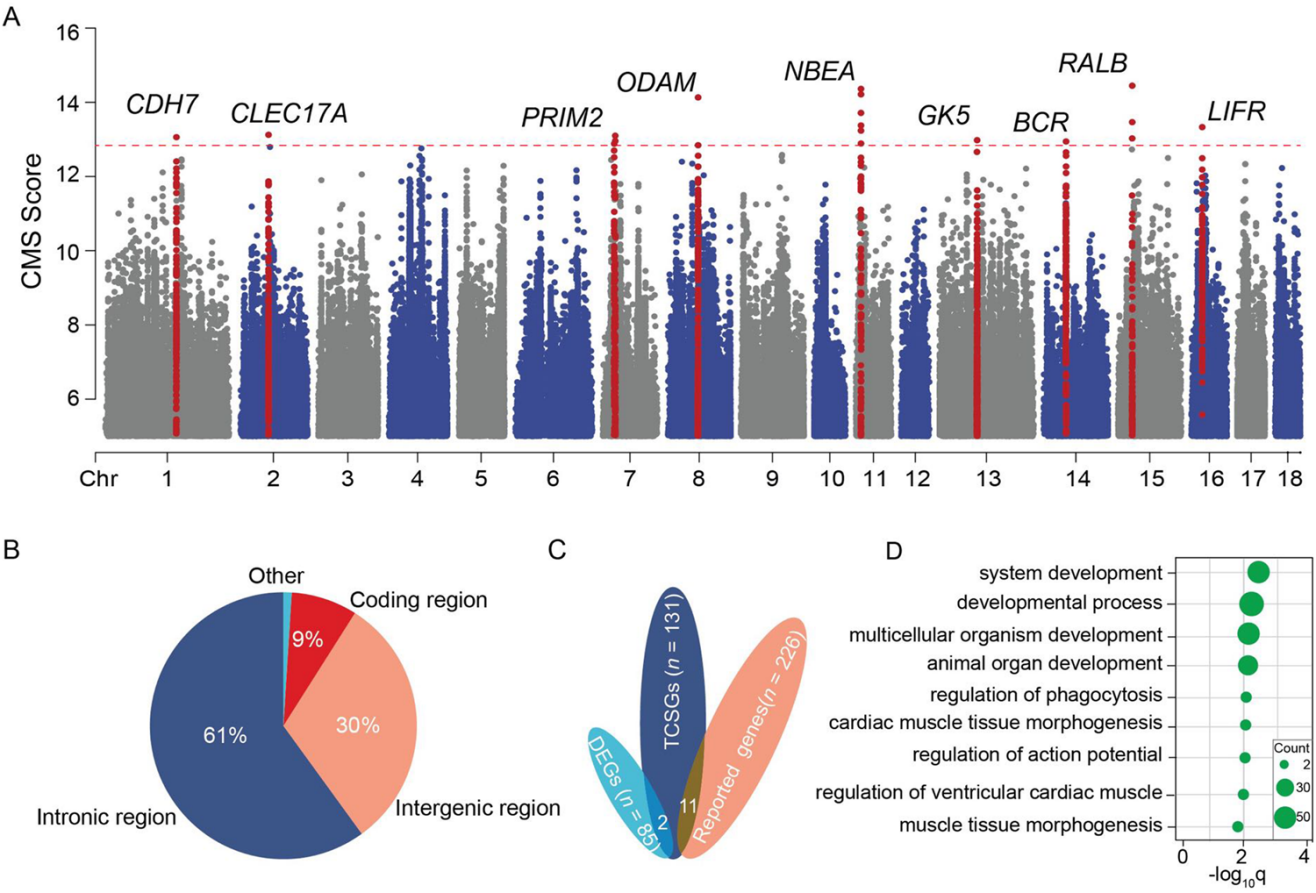
Genome-wide scanning for genetic signatures of TBPs. (**A**) The distributions of TCSGs in the TBP genome. The top 10 TCSGs at the genome-wide significant loci are highlighted in red. (**B**) Annotation of the 4,598 SNPs with a CMS > 1‰ (8.82) by VEP. (**C**) Venn diagram of the TCSGs, published genes and DEGs. (**D**) KEGG and GO enrichment analyses by g: Profiler. The gene counts are represented by circle size.

We identified 4,598 SNPs selected in TBP (referred to as TBPs candidate SNPs (TCSSs)), which were involved in the selection of 131 TBP candidates (TCSGs), including 121 novel genes and 11 reported genes. *SORCS3* and *CD36* are differential expression genes (DEGs) in the lungs of TBPs^21^(Supplementary Table 4). In particular, among the top 10 TCSGs, 2 genes, *BCR* and *ODAM,* were reported in previous studies^3–5^, though the other 8 genes, *RALB*, *NBEA*, *LIFR*, *CLEC17A*, *PRIM2*, *CDH7*, *GK5* and *FAM83B*, were novel signal in our study, suggesting that the vast majority of the genes were novel candidates for TBPs (Table 1; Fig. 2A, C, Supplementary Fig. 2). We conducted functional annotation for 4,598 TCSSs, and the results revealed that 91% of the TSCSs were located in noncoding regions; among them, 535 SNPs were located in regulatory regions. In addition, we identified 413 (9%) TSNSs in coding regions (Fig. 2B, Supplementary Table 3). We found that 3 missense genes were enriched in TBPs. The novel top Tibetan-enriched missense gene was rs343993998 (CMS = 9.04, *F*_ST(TBPs-Duroc)_ = 0.52, *F*_ST(TBPs-YKX)_ = 0.57) in *PRR14L* (a protein coding gene highly expressed in the lung), which was reported to be associated with the Forced Expiratory Volume In 1s / Forced Vital Capacity (FEV1/FEC) ratio^26^and is likely involved in improving lung function in response TBP HAA (Fig. 2B, Supplementary Table 5).

**Table 1:** The top 10 TCSGs among the TBPs.

Chromsome	SNPs	Position (*Sus scrofa* 11.1)	Genotype	TBPEs	CMS	*F*_ST_	Genes	TBPs (*n* = 60)	DU (*n* = 25)	YKX (*n* = 16)
15	rs324091221	31078547	A > T	T	14.45	0.40	RALB	0.83	0.35	0.43
11	rs344284117	11145093	A > G	G	14.03	0.41	NBE.4	0.56	0.04	0.17
16	rs700613046	23681563	C > A	A	13.33	34	LIFR	0.92	0.55	0.63
2	rs326031478	64694210	T > C	C	13.12	0.49	CLEC17A	0.97	0.27	0.37
7	rs325487910	28315938	A > G	G	13.1	0.66	PR1M2	0.88	0.17	0.27
1	rs343112528	156356296	G > A	A	13.06	0.57	CDH7	0.66	0.01	0.07
13	rs321211870	83019802	G > A	A	12.97	0.11	GK5	0.18	0.01	0,01
14	rs339107125	49301899	A > T	T	12.95	0.57	BCR	0.98	0.27	0.50
7	rs343496527	26125849	T > C	C	12.88	0.61	FAM83B	0.93	0–35	0.82
8	rs324521285	67065076	A > G	G	12.84	0.33	ODAM	1.00	0.75	0.89

TBPEs are an alleles that are frequently enriched in TBPs.

For 131 TCSGs, KEGG and GO enrichment analyses were performed by g: Profiler, and the results revealde that 38 genes were involved in system development (GO: 0048731, *q* = 0.003), animal organ development (GO: 0048513, *q* = 0.006) and multicellular organism development (GO: 0007275, adjusted *q* = 0.006). In addition, multiple genes were enriched in cardiac muscle tissue morphogenesis (GO: 0055008, *q* = 0.03) and muscle tissue (GO: 0060415, *q* = 0.04) among the TBPs, which suggesting that TCSGs likely perform different functions in multiple organs (Fig. 2D; Supplementary Table 6).

### Associations of TCSs with adaptive phenotypes in TBPs

**Figure 3:**
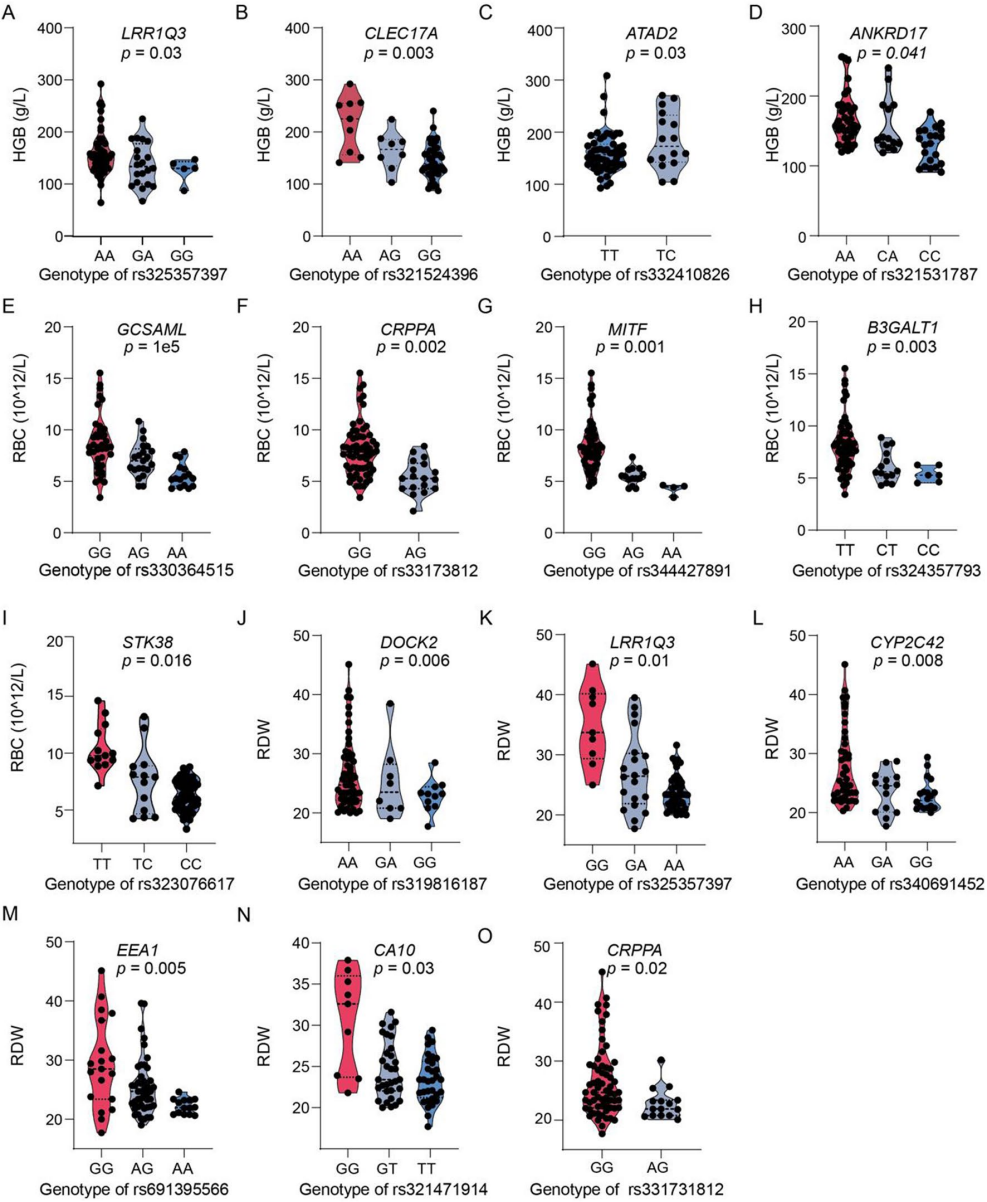
Correlation analysis of TCSSs and adaptive phenotypes in TBPs. (**A**–**D**) Hemoglobin levels of different genotypes of 4 gene variants in 137 TBPs; (**E**–**I**) Red blood cell counts of different genotypes of 5 gene variants in 137 TBPs; (**J**–**O**) Red blood cell distribution width levels of different genotypes of 6 gene variants in 137 TBPs. *p* is the adjusted *p* value (100,000 permutation tests).

To further reveal the contribution of TCSGs to HAA in TBPs, we conducted a genome-wide association analysis of TBPs with adaptive phenotypes. A linear additive model was applied, and we found that 29 TCSSs involved in 13 genes (*LRRIQ3*, *CLEC17A*, *ATAD2*, *B3GALT1*, *GCSAML*, *CRPPA*, *MITF*, *CA10*, *DOCK2*, *CYP2C42*, *EEA1, STK38,* and *ANKRD17*) were associated with at least one adaptive trait after 100,000 permutation tests (Fig. 3; Supplementary 7). One of the top ten TCSGS genes, *CLEC17A* (C-Type Lectin Domain Containing 17A), is a protein coding gene related to mannose binding and fucose binding^27^. The peak-SNP (rs326031478, CMS = 13.12, XPEHH = 2.2, *F*_ST(TBPs-Duroc)_ = 0.495, *F*_ST(TBPs-YKX)_ = 0.458, iHS = 4.1) showed a remarkable selection signature and revealed a particularly distinctive LD decay pattern in TBPs compared to other pigs (Fig. 3A; Table 1). We identified multiple TCSSs of *CLEC17A* that were associated with RBCs and HGB; for example, rs343477882 (adjusted *p* = 0.006, beta = −2.12) relative to HGB and RBCs had a CMS of 11.81, and rs319060986, located in the 3'UTR of *CLEC17A,* had a CMS of 10.56. We speculated that *CLEC17A* might play an important role in protecting against excessive HGB and RBC proliferation in TBPs. The adaptive alleles in *GCSAML* showed strong associations with reduced red blood cell counts (RBCs, adjusted *p* = 1e5, beta = −1.5, CMS = 8.84) (Fig. 3E, Supplementary Table 7). *EEA1* (early endosome antigen 1) is involved in activated TLR4 and cytoskeletal signaling^28,29^and harbors a series of SNPs that are strongly associated with RBCs and RDW (Fig. 3M, Supplementary Table 7). On average, each SNP explained 1.46% (0.438 g/L), 4.4% (0.12 × 10^12/L) and 2.9% (0.13) of the variance in HGB, RBC and RDW levels, respectively, indicating that the lower HGB and RBC levels in TBPs might result from the high Tibetan-specific alleles of TCSSs.

### Polygenic effects of genetic adaptation in TBPs

**Figure 4:**
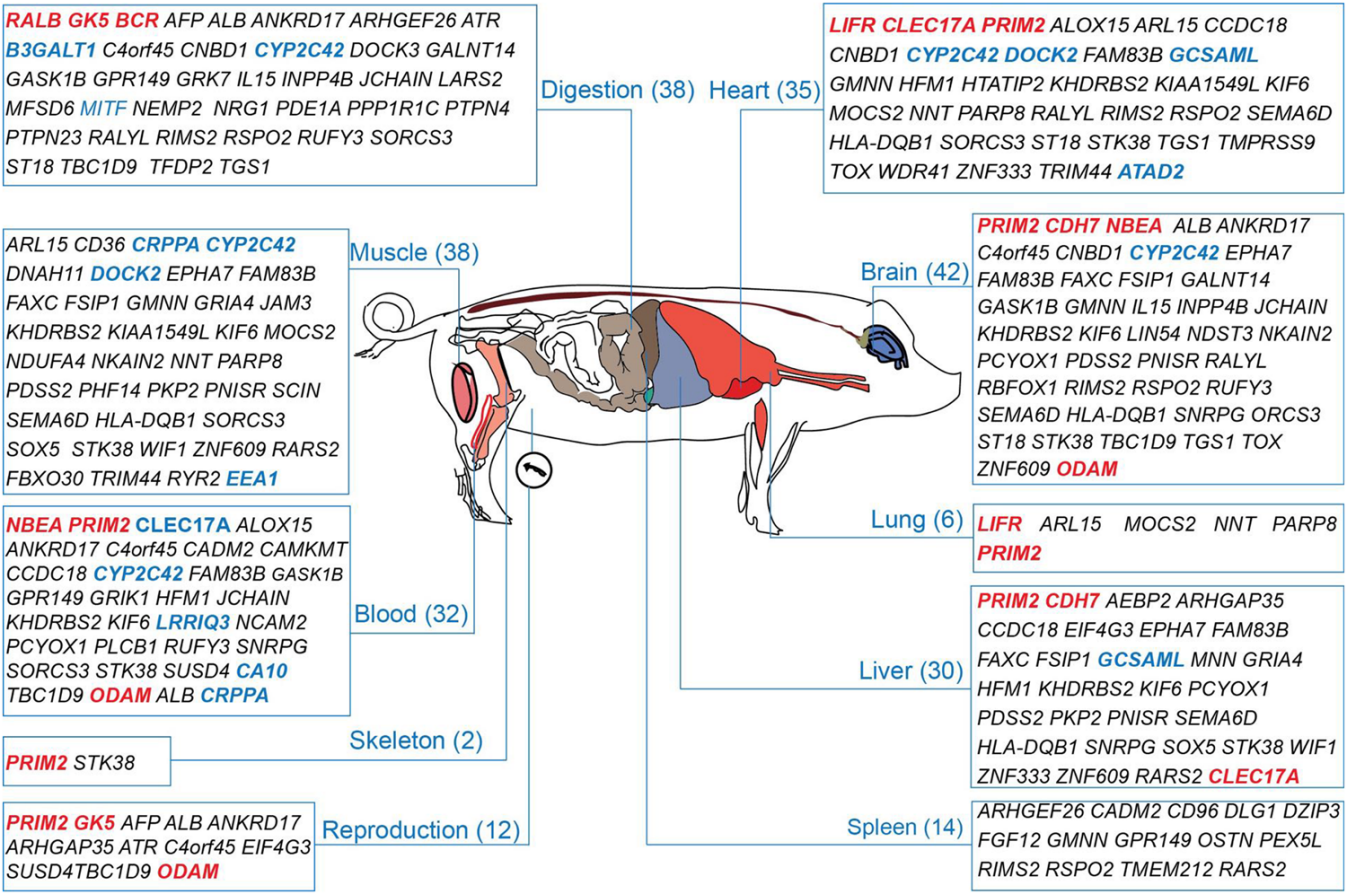
Enrichment of 131 TCSGs across organs/systems. The organs/systems, enriched genes and total counts in each organ/system are denoted. Red: top ten TCSGs; Blue: association of TCSGs with the blood phenotype.

To investigate the phenotypic effects of TCSGs and links to the body parts they affect, PigQTLdb (https://www.animalgenome.org/QTLdb/app) was used to establish possible relationships between TCSGs and organs/systems. The analysis revealed that these TCSGs function in multiple organs/systems, such as blood (30 genes), heart (35 genes), brain (42 genes), muscle (38 genes), digestion (38 genes), and liver (30 genes) (Fig. 4). Interestingly, a series of TCSGs seem to work in multiple organs/systems and have pleiotropic effects on regulating the physiological adaptation of TBPs. For example, *PRIM2*, one of the top 10 TCSGs, functions in multiple organs/systems (Fig. 4) due to its role in the initiation of DNA synthesis^30,31^. Similarly, *ODAM* appears in 3 different organs/systems and likely plays a role in odontogenesis and tooth generation ^32^.

Strikingly, 42 TCSGs are linked to the brain, including 3 of the top ten genes (*PRIM2, CHD7,* and *ODAM*), *CDH7* (cadherin 7) and *PRIM2* (DNA primase subunit 2), which are known to function in the growth potential of PN axons^33^and insomnia^34^. Furthermore, 35 TCSGs related to the heart likely contribute to the ability of TBPs to maintain greater and stronger heart function. Compared to that of LDPs, better heart function is one of the adaptive phenotypes of TBPs^21^, providing an alternative gene base for TBPs with better heart function. Another important system is the blood circulation, which carries O_2_ and nutrients to organs; 30 TCSGs are involved in the blood. Many mammals and birds, such as Tibetans^6,35^, Ochotonidae^36^, dogs^37^, horses^1^and bar-headed geese^38,39^, adapt to HAA by transforming the O_2_ transportation capacity of hemoglobin. *LIFR* (leukemia inhibitory factor receptor) and *PRIM2* are known to promote tumor growth, metastasis, and angiogenesis and increase angiogenic activity and coronary artery disease^40^. In particular, 13 TCSGs were associated with adaptive blood phenotypes (HGB, RBC, RDW) (Fig. 3A–O) and *CLEC17A* (C-Type Lectin Domain Containing 17A) among the top 10 TCSGs and were related to reduced HGB (adjusted *p* = 0.003) (Fig. 3B). These findings support our association results.

Overall, based on 131 functionally chunked TCSGs, we concluded that genes under selection in TBPs were subjected to various physiological systems, suggesting that TBP adaptation to high-altitude environments involves multigene interactions and multiple organ or system-level adaptation processes.

## Discussion

Adaptive phenotypes have been systematically characterized in Tibetans^41^. Phenotypic studies of TBPs are poorly understood, and the adaptive phenotypes of TBPs are not clear. Therefore, we surveyed 9 blood indices across different altitudes and found that HBG, RBC, and RDW were significantly different between TBPs and LDPs (Fig. 1B, C). Most importantly, the patterns of HGB and RBCs are similar to the adaptation strategy of Tibetans^6,41^ and indicate a blunted regulatory mechanism to maintain hemoglobin levels in a relatively normal range; conversely, hyperhemoglobinemia and polycythemia are incidental to LDPs. Regulating hemoglobin is a commonly employed strategy for avoiding HAA in plateau mammals^1,36,37^. The hemoglobin concentration of TBPs is significantly lower than that of LDPs at high altitudes; however, the HGB is higher than that of LDPs (living at low altitudes) (Fig. 1C); Decreased hemoglobin was also found in Tibetans at high altitudes^14^, which is consistent with the results in Tibetan pigs in this study. However, studies have also shown that plateau Sherpas have unique adaptations, which shifted the focus from hemoglobin concentration to hemoglobin mass and plasma volume, which may affect altitude adaptation^16^. The only reason for our inconsistent results could be that different species have different mechanisms of adaptation to the plateau environment^17^. Therefore, the hemoglobin measurement standard developed at low altitudes (< 2500 m) is no longer suitable for TBPs. We proposed a reference range for hemoglobin concentrations in TBPs of 158.9 ± 68.9 based on our phenotypic data, which will be helpful for diagnosing TBPs and raising them scientifically. In this study, we identified potential alternate causal genes, and highly credible TBP-positive selected genes by measuring the blood phenotypes of TBPs and LDPs at different altitudes. These findings will be valuable resources for future HAA studie in TBPs.

Based on our sample and data, we generated a robust positive selection gene set for TBPs. Primarily, we employed the CMS method (see Methods) to scan the underlying naturally selected genes. Compared to other single methods, this approach overcomes methodological bias. However, this strategy may miss weakly selected. Another limitation defect is that we only annotated the peak SNP-located genes as TCSGs, and the other linkage genes were excluded. For example, in the *RALB* gene region (940.2 kb), 5 genes (*EPB41L5*, *INHBB*, *PTPN4*, *TMEM177*, *TMEM185B*) were linked to *RALB*, and the peak SNP (rs343550189, CMS = 14.45) was within *RALB* to maintain the robustness of the genes; therefore, we preserved genes that harbored the peak SNP as TCSGs (Supplementary Table 8). Ultimately, we obtained a highly credible gene set including 131 TCSGs. We identified 13 TCSGs, including one of the top10 genes (*CLEC17A*), and the association analysis revealed that the adaptive allele of *CLEC17A* (adjusted p = 0.03) is responsible for reducing HGB (Fig. 3). Similarly, the SNP rs330364515 (adjusted p = 1e5) in *GCSAML* accounts for RBCs, and large-scale human population GWA analysis revealed that *GCSAML* is responsible for RBCs, platelet count and mean platelet volume^42^. In addition, of the top 10 TCSGs, *LIFR* (CMS = 14.45) is a polyfunctional cytokine that is involved in cellular proliferation, differentiation, and embryonic development^43^. Large-scale population GWAs showed that *PRIM2* (CMS = 13.1) is related to coronary artery disease and myocardial infarction^40^, *CDH7* (CMS = 13) is associated with feed efficiency, body mass index and body height in pigs^44^; *GK5* (Glycerol K1inase;CMS = 12.97) is related to eosinophil count and mean corpuscular hemoglobin (MCH); *NBEA* (Neurobeachin; CMS = 14.36) encodes a member of A-kinase anchor proteins to target the activity of protein kinase A to specific subcellular sites and is associated with neurodevelopmental disorder^45^; and *FAM83B* (Family with sequence similarity 83, member (B) has been reported as an important intermediary in EGFR/RAS signaling and is related to cellular differentiation and proliferation^46^. These TCSGs might be responsible for the adaptive phenotypes of lower HGB and better lung and heart function.

Hypoxia adaptation in Tibetans is dominated by two genes (*EPAS1 and EGLN1*). In this study, we did not detect the strong selection of *EPAS1* and E*GLN1* among the TBPs, possibly due to the limited sample size. We cannot rule out the possibility that *EPAS1* and *EGLN1* were positively selected among the TBPs; and large-scale whole-genome data are needed for verification. However, it is possible that TBPs may have adapted to hypoxia through the contributions of other genes. Different animals have different strategies to adapt to enviroment on the the plateau^6^.

To understand the functions of the TCSGs alternating with those of HAA in TBPs, we linked the genes to the organs/systems. In our study, 32 TCSGs were involved in the blood system, of which 5 genes were associated with blood traits (Fig. 3A, B, F, L, N). The blood system is sensitive to hypoxia, and *EPAS1* and *EGLN1* are two star genes that reduce HGB and RBCs to help Tibetans avoid HAA and prevent polycythemia^6,35,41^. Here, we found that multiple TCSGs are related to HGB, RBCs, and RDW. The robust Darwinian positive selection gene *CLEC17A* is associated with greduced HGB, which might be a candidate causal gene set for blood traits regulating HAA in TBPs. In addition, we found that more than 30 genes in the TBP cardiopulmonary system, such as *PRIM2, KHDRBS2* and *ARL15* (GTPase15), are known to be functionally involved in coronary artery disease, the FEV/FVC ratio and cardiac septum development^40,47,48^, indicating that these TCSGs might play important roles in the heart and lung function of TBPs.

## Conclusions

In summary, 131 TCSGs are involved in TBP HAA. Our results suggest that HAA in TBPs is a multigene interaction process and is associated with a wide range of complex traits and a variety of biological processes. Therefore, the combination of large-scale WGS and full-scale phenotypic data is needed to reveal the genetically imprinted genes and adaptive phenotypes, including embryonic development, morphogenesis, cardiopulmonary function, skeletal muscle, lipid metabolism and reproduction. Overall, we provide a robust TCSG set as a genetic basis that will be useful for further TBPs studies.

### Supplementary Information

Supplementary Figures.

Supplementary Tables.

## Data Availability

All reported raw the chip data with this article have been deposited into the China National Center for Bioinformation database (https://bigd.big.ac.cn/gvm/getProjectDetail?Project=GVM000695) under accession number GVM000695. All other data supporting the results of this study are available in this paper and its supplementary tables. Other data sets associated with this article, from the national center for biotechnology information database (https://www.Ncbi.nlm.nih.gov), the registration numbers are SRA096093, CRA001606, PRJEB1683, PRJNA186497, PRJNA260763.
