# Supplementary material for: Genetic characterization of Tibetan pigs adapted to high altitude under natural selection based on a large whole-genome dataset
Source: Sci Rep. 2024 Jul 24;14:17062. doi: 10.1038/s41598-024-65559-3 (PMC11269713; doi:10.1038/s41598-024-65559-3)
Supplement: Supplementary file 1 — Supplementary Figures. [file 41598_2024_65559_MOESM1_ESM.docx]

**
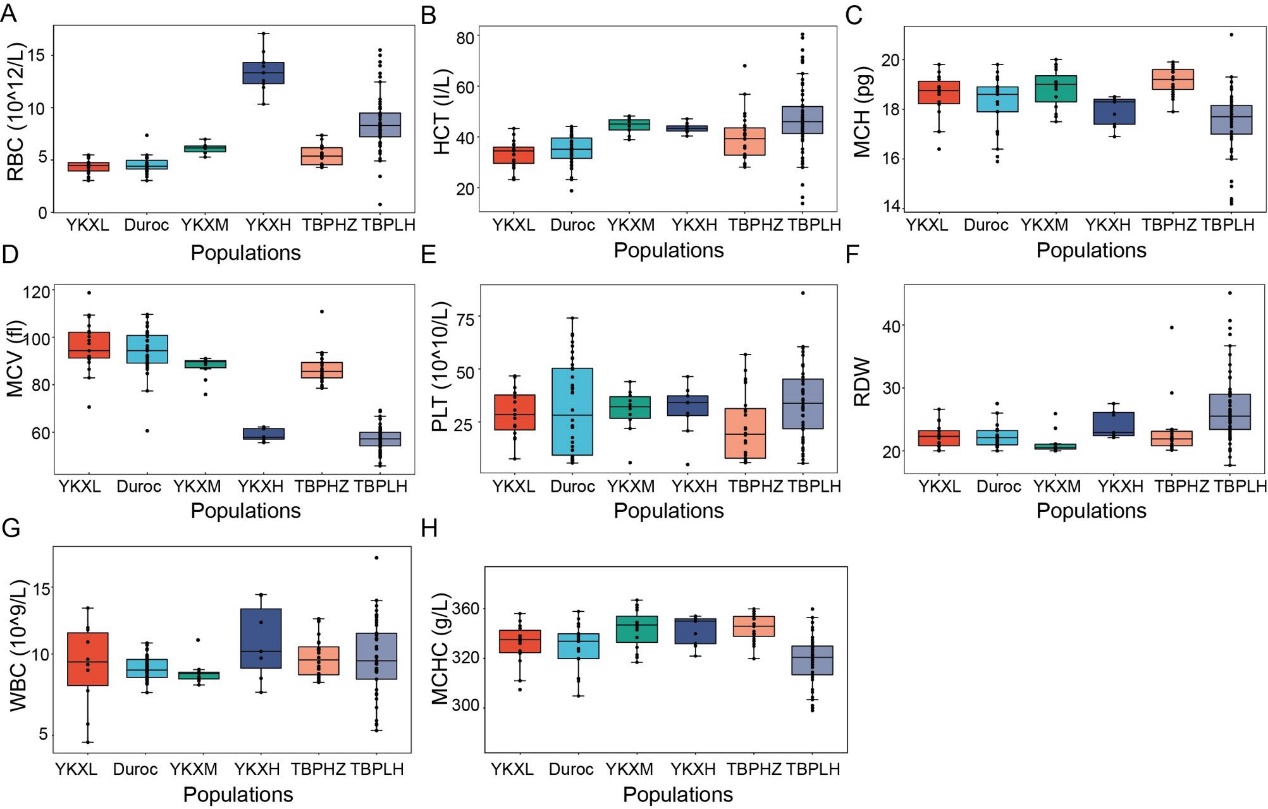
**

**Supplementary Figure 1** Eight-component phenotypic analysis. **A.** RBC, and RBC were significantly different between TBP and LDP (YKX, Duroc); **B**. HCT; **C.** MCH**; D.** MCV**; E.** PLT**; F.** RDW and RDW were significantly significant different between TBP and LDP (YKX, Duroc); G. WBC; **H.** MCHC; YKXL: Yorkshire from lower altitudes (<1,000 m); YKXM Yorkshire from middle altitudes (1,000 <YKXM <3,600 m); YKXM Yorkshire from high altitudes (>3,600 m).


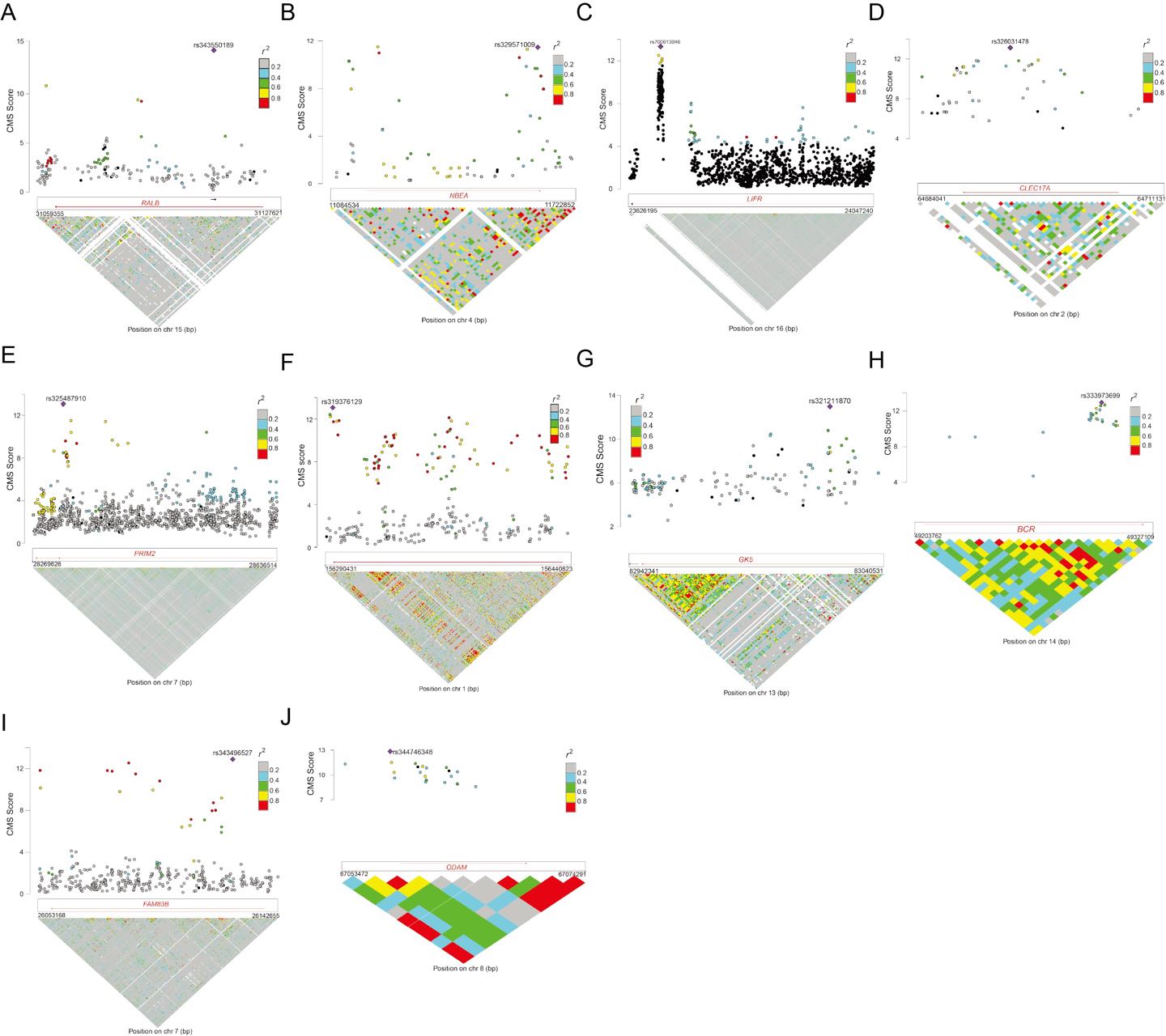


**Supplementary Figure 2** Regional plot of the top 10 TCSGs**.** The SNP rsID number and CMS of the most significant peak SNP are labeled on top of the SNP. The r^2^ LD was estimated using 69 TBPs.
